# Short-Term Stability of the Fecal Microbiota in Wild *Cavia tschudii* During Early Captivity Transition

**DOI:** 10.3390/microorganisms14081629

**Published:** 2026-07-26

**Authors:** Hugo Frías, Juan José Barrios, Lizeth A. Heredia-Vilchez, Paul Fernandez-Castro, Nilton Luis Murga Valderrama, Segundo Melecio Portocarrero Villegas, Angelo V. Esparraga-Calle, Gleni Tatiana Segura Portocarrero, Jorge L. Maicelo Quintana, Lamberto Valqui-Valqui, Leandro Valqui, Miguel Ángel Arista Ruiz, Segundo José Zamora Huamán, Rainer M. López Lapa, William Bardales Escalante, José Américo Saucedo-Uriarte

**Affiliations:** 1 Facultad de Ingeniería Zootecnista, Biotecnología, Agronegocios y Ciencia de Datos (FIZBAC), Universidad Nacional Toribio Rodríguez de Mendoza de Amazonas (UNTRM), Chachapoyas 01001, Amazonas, Peru; hugo.frias@untrm.edu.pe (H.F.); 7144768721@untrm.edu.pe (J.J.B.); jmaicelo@untrm.edu.pe (J.L.M.Q.); jose.zamora@untrm.edu.pe (S.J.Z.H.); jose.saucedo@untrm.edu.pe (J.A.S.-U.); 2 Instituto de Investigación en Ganadería y Biotecnología (IGBI), Facultad de Ingeniería Zootecnista, Biotecnología, Agronegocios y Ciencia de Datos (FIZBAC), Universidad Nacional Toribio Rodríguez de Mendoza de Amazonas (UNTRM), Chachapoyas 01001, Amazonas, Peru; paul.fernandez@untrm.edu.pe (P.F.-C.); 7353415481@untrm.edu.pe (A.V.E.-C.); miguel.arista.epg@untrm.edu.pe (M.Á.A.R.); william.bardales@untrm.edu.pe (W.B.E.); 3 Laboratorio de Biotecnología Animal, Reproducción y Mejoramiento Genético (BIOLAB), Instituto de Investigación en Ganadería y Biotecnología (IGBI), Facultad de Ingeniería Zootecnista, Biotecnología, Agronegocios y Ciencia de Datos (FIZBAC), Universidad Nacional Toribio Rodríguez de Mendoza de Amazonas (UNTRM), Chachapoyas 01001, Amazonas, Peru; nmurga.fizab@untrm.edu.pe (N.L.M.V.); tatiana.segura@untrm.edu.pe (G.T.S.P.); 4 Programa de Doctorado en Ciencias para el Desarrollo Sustentable, Universidad Nacional Toribio Rodríguez de Mendoza de Amazonas (UNTRM), Chachapoyas 01001, Amazonas, Peru; segundo.portocarrero@untrm.edu.pe; 5 Laboratorio de Agrostología, Instituto de Investigación en Ganadería y Biotecnología (IGBI), Facultad de Ingeniería Zootecnista, Biotecnología, Agronegocios y Ciencia de Datos (FIZBAC), Universidad Nacional Toribio Rodríguez de Mendoza de Amazonas (UNTRM), Chachapoyas 01001, Amazonas, Peru; lamberto.valqui@untrm.edu.pe (L.V.-V.); leandro.valqui.epg@untrm.edu.pe (L.V.); 6 Laboratorio de Fisiología Molecular (FISIOLAB), Instituto de Investigación en Ganadería y Biotecnología (IGBI), Facultad de Ingeniería Zootecnista, Biotecnología, Agronegocios y Ciencia de Datos (FIZBAC), Universidad Nacional Toribio Rodríguez de Mendoza de Amazonas (UNTRM), Chachapoyas 01001, Amazonas, Peru; rainer.lopez@untrm.edu.pe

**Keywords:** fecal microbiota, captivity transition, 16S rRNA sequencing, microbial diversity, host–microbiome interactions, *Cavia tschudii*

## Abstract

This study evaluated the short-term response of the fecal microbiota of wild *Cavia tschudii* during its transition from natural conditions to captivity under a standardized alfalfa-based diet. Fecal samples were collected over a 21-day period and analyzed using 16S rRNA gene sequencing to assess microbial diversity, community structure, and taxonomic composition. Rarefaction analysis indicated sufficient sequencing coverage; however, samples were normalized to 500 reads per sample, and results should be interpreted cautiously. No significant changes were detected in alpha diversity, beta diversity, or dominant taxonomic composition across sampling time points. The microbiota was consistently dominated by Bacteroidota and Firmicutes, accounting for more than 85% of total relative abundance. Minor taxa such as Methanobacteriales and Spirochaetales were present at low and stable abundances. Despite differences between natural forage and the alfalfa-based diet, microbial community structure showed limited temporal variation during the study period. The absence of detectable parasitic infections further supports that observed patterns reflect host–microbiota dynamics under controlled conditions. Functional predictions suggested variation in metabolic pathways but should be interpreted cautiously due to the limitations of 16S rRNA-based inference. Overall, these findings suggest limited temporal variation in the fecal microbiota of *Cavia tschudii* during the first three weeks of captivity under the conditions evaluated. However, due to the exploratory nature of the study and its methodological limitations, these results should be interpreted with caution. This study provides baseline insights into limited temporal variation in the fecal microbiota of a wild caviid species and contributes to understanding host–microbiome interactions relevant to conservation and potential domestication processes.

## 1. Introduction

The host-associated microbiota has become a central component in studies of animal and human health due to its role in metabolic regulation, immune modulation, and overall physiological homeostasis [1,2]. In animal systems, fecal microbiota profiling is widely used as a non-invasive approach to characterize gut microbial communities, particularly in longitudinal studies assessing environmental and dietary transitions. In herbivorous mammals, the gastrointestinal microbiota plays a fundamental role in the degradation of plant-derived substrates and the production of metabolites essential for host nutrition. Beneficial bacterial genera such as *Lactobacillus* and *Bifidobacterium* have been associated with immune regulation and maintenance of intestinal barrier integrity [3,4,5]. Likewise, fibrolytic taxa such as *Prevotella* and *Ruminococcus* contribute to the breakdown of complex polysaccharides and the production of short-chain fatty acids, supporting energy metabolism and feed efficiency in herbivorous hosts [6]. These microbial processes are essential in hindgut fermenters, where complex microbial communities enable efficient utilization of fibrous diets [7]. Furthermore, gut microbial diversity is shaped by host diet, evolutionary history, and ecological niche, linking microbial composition with host adaptation across mammals [8].

In livestock and managed animal systems, fecal microbiota analysis has become an important tool for monitoring animal health, welfare, and productivity [9]. Studies in productive species have demonstrated that gut microbial composition is closely related to digestive efficiency, immune function, and overall performance [10]. In addition, dietary interventions and management practices can significantly modify microbial communities in species such as guinea pigs and poultry, reinforcing the importance of microbiome research in animal production systems. Beyond domestic species, captivity and domestication processes introduce abrupt changes in diet, habitat, and microbial exposure, which may alter gut microbial communities. Ecological shifts associated with domestication have been shown to significantly influence gut microbiota composition in mammals [11]. However, the short-term dynamics of these changes, particularly during the initial transition from wild to captive conditions, remain poorly understood.

*Cavia tschudii* Fitzinger, 1867 [12], commonly known as the mountain guinea pig or “sacha cuy,” is a wild rodent native to the Peruvian Andes and phylogenetically related to the domestic guinea pig (*Cavia porcellus*) [13]. In its natural habitat, this species feeds primarily on native grasses and herbaceous vegetation, contributing to ecosystem dynamics while serving as prey for native predators. Due to its genetic proximity to domestic guinea pigs, *Cavia tschudii* has attracted increasing interest in conservation, captive management, and potential productive systems. However, individuals maintained under captive or semi-captive conditions are exposed to controlled diets and environments that differ markedly from those found in the wild, which may influence gut microbial composition and functionality. Despite the growing interest in microbiota research, little is known about the early response of gut microbial communities in wild caviids during the transition from natural habitats to captivity. In particular, it remains unclear whether short-term environmental and dietary changes are sufficient to disrupt microbial diversity and community structure, or whether these communities show limited temporal variation during the early stages of adaptation.

Therefore, this study aimed to evaluate the temporal response of the fecal microbiota of *Cavia tschudii* during the first three weeks following its transition from wild conditions to captivity under a standardized alfalfa-based diet. We hypothesized that microbial diversity and community structure would show limited short-term variation, reflecting the capacity of the gut microbiota to maintain its overall composition during early captive adaptation.

## 2. Methodology

### 2.1. Experimental Animals and Study Design

Individuals of *Cavia tschudii* were captured in the Sallca Annex, Limabamba District, Rodríguez de Mendoza Province, Amazonas, Peru (6°29′33.0″ S, 77°31′33.6″ W). Capture was carried out in collaboration with local residents by progressively enclosing the area to minimize environmental disturbance.

**Figure 1:**
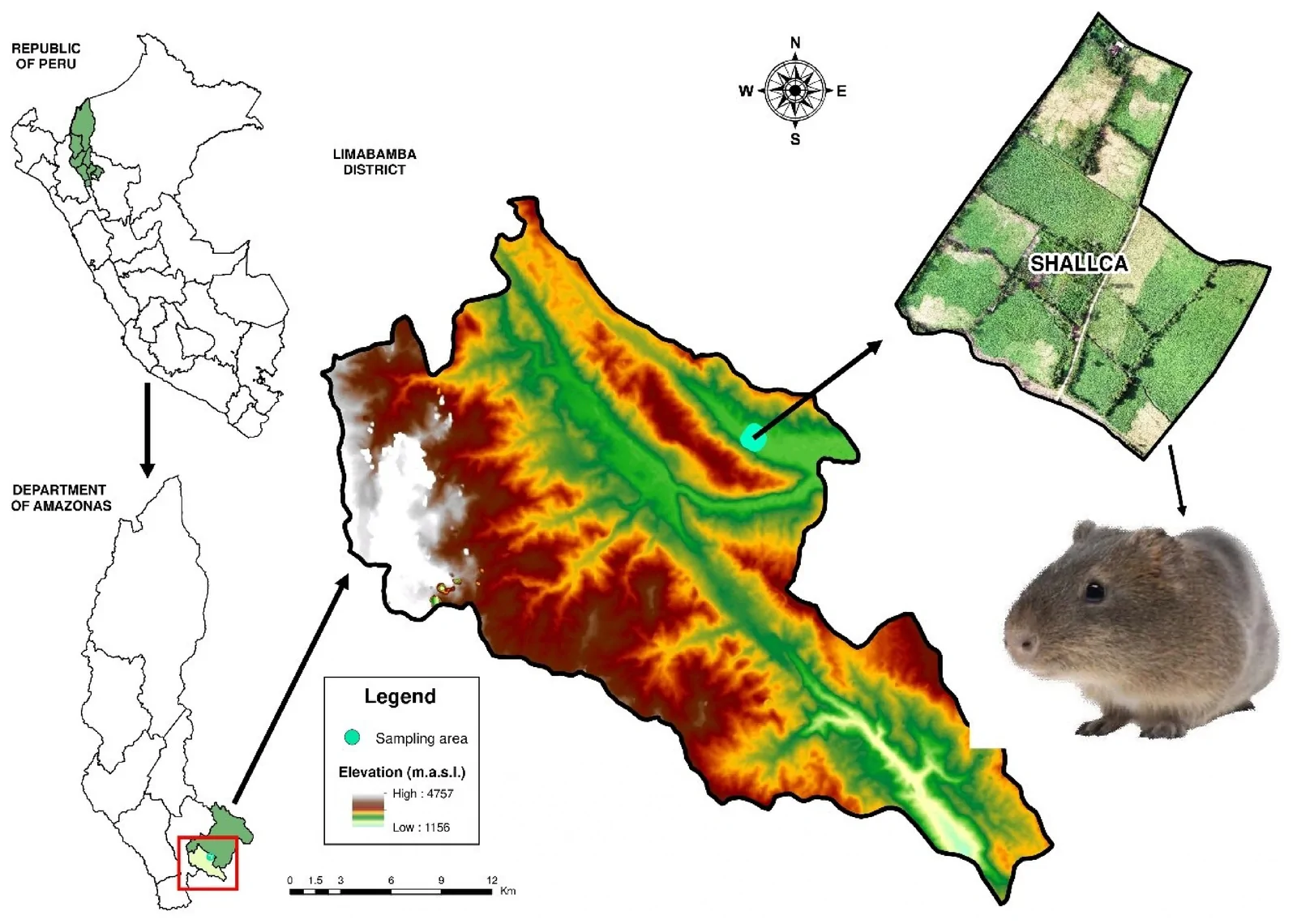
Location map of the collection area of *Cavia tschudii* in the Sallca Annex, Limabamba, Rodríguez de Mendoza, Amazonas.

Captured animals were transported to the Chachapoyas Experimental Station (6°13′59″ S, 77°51′11″ W) of the Universidad Nacional Toribio Rodríguez de Mendoza de Amazonas (UNTRM) (Figure 1). Individuals were selected based on good physical condition, absence of parasitic infection, and morphological characteristics consistent with the species. Adult status was determined based on body weight (~320 g) and fully developed morphological characteristics consistent with the species.

Of the eight individuals captured, five met the inclusion criteria and were included in the study. The use of adult individuals was intentional, as fecal microbiota composition is generally more stable after maturation.

A parasitological assessment was conducted prior to microbiota analysis to exclude individuals with parasitic infections. Approximately 5 g of fecal material per individual was analyzed using flotation and sedimentation techniques at the Laboratory of Infectious and Parasitic Diseases of Domestic Animals (LABISAN, UNTRM, SL01LA12). Ectoparasite screening was performed through direct examination of fur and skin using a stereoscopic lens and combing. Only parasite-free individuals were included in subsequent analyses to reduce potential confounding effects.

Prior to admission to captivity, all selected individuals received external antiparasitic treatment using a commercial formulation containing cypermethrin. The product was applied topically following manufacturer recommendations. Animals were monitored individually until the product had dried to prevent grooming and minimize accidental ingestion.

This study followed a longitudinal repeated-measures design in which the same five individuals were sampled at four consecutive time points: on the day of capture, immediately before captivity (Day 0), and after 7, 14, and 21 days under captive conditions.

At the time of capture (Day 0), individuals were living under natural conditions and feeding primarily on native grasses, including *Brachiaria mutica*. Fecal samples collected at this time point were considered representative of the natural grazing condition.

After transport, animals were housed individually in enclosures (1.5 × 1 × 1 m) with straw bedding and shelters made from dried *Agave americana* logs. Facilities were disinfected 15 days prior to animal arrival using a commercial disinfectant solution.

During captivity (Days 7, 14, and 21), all individuals were fed a standardized diet consisting exclusively of fresh alfalfa (*Medicago sativa*), provided twice daily (8:00 a.m. and 4:00 p.m), with water available ad libitum. No experimental dietary treatments were applied; rather, the study evaluated the natural transition from wild grazing conditions to a controlled captive diet.

The experimental protocol was approved by the Institutional Research Ethics Committee of UNTRM (CIEI-No. 00119) and conducted in accordance with Peruvian animal welfare regulations (Law No. 30407) and international guidelines.

### 2.2. Bromatological Analysis

Bromatological analyses were performed for both *Brachiaria mutica*, representing the main natural forage consumed under wild conditions, and *Medicago sativa*, corresponding to the standardized captive diet. These analyses were conducted to characterize the nutritional profile of both forage sources and to provide contextual information for interpreting potential fecal microbiota changes associated with the transition from natural grazing to captivity.

Analyses were conducted at the Animal Nutrition and Bromatology Laboratory (LABNUT, UNTRM) following AOAC standard methods [14,15] and ANKOM Technology (Macedon, NY, USA) [16] procedures. The following parameters were determined: moisture, ash, crude protein (Kjeldahl method), ether extract, neutral detergent fiber (NDF), acid detergent fiber (ADF), acid detergent lignin (ADL), and nitrogen-free extract (Supplementary Table S1).

### 2.3. Fecal Sample Collection and DNA Extraction

Fresh fecal samples were collected immediately after defecation using sterile swabs and transferred into sterile tubes containing Amies transport medium. Samples were transported under refrigerated conditions and processed within one week.

Total microbial DNA was extracted using the Quick-DNA™ Fungal/Bacterial Miniprep Kit (Zymo Research, Irvine, CA, USA), following the manufacturer’s instructions. DNA quality and concentration were assessed using a NanoDrop spectrophotometer (Thermo Fisher Scientific, Waltham, MA, USA) and a Qubit fluorometer (Thermo Fisher Scientific, Waltham, MA, USA).

### 2.4. 16S rRNA Gene Amplification, Sequencing and Taxonomic Assignment

The V4 hypervariable region of the bacterial 16S rRNA gene was amplified using the primers 515F 5′-GTGCCAGCMGCCGCGGTAA-3′ and 806R 5′-GGACTACHVGGGTWTCTAAT-3′. Amplicon libraries were sequenced on the Illumina MiSeq platform (Illumina, San Diego, CA, USA) with paired-end reads (2 × 150 bp), generating fragments of approximately 250 bp.

Raw sequence data were processed using the nf-core/ampliseq v2.9.0 pipeline [17], executed with Nextflow v4.2.1 [18]. Quality assessment was performed using FastQC v0.12.1 and MultiQC v1.26 [19,20], and adapter trimming was conducted using Cutadapt v5.0 [21].

Amplicon sequence variants (ASVs) were inferred using DADA2 v1.26.0 [22] within the QIIME 2 framework [23]. Taxonomic classification was performed against the SILVA v138.2 database [24]. Non-bacterial sequences (chloroplast, mitochondrial, and archaeal) were removed prior to downstream analyses.

Samples were rarefied to a sequencing depth of 500 reads per sample, corresponding to the lowest retained depth across samples. This normalization was applied to standardize sampling effort; however, given the relatively low sequencing depth, diversity estimates were interpreted cautiously and primarily as exploratory indicators.

For taxonomic barplot visualization, the ggbartax function implemented in the MicrobiotaProcess R package was used with the parameter topn = 5, which displays the five most abundant taxa across all samples while grouping the remaining taxa into the category “Others”. In family- and genus-level plots, placeholder or unclassified taxa lacking informative taxonomic annotation were excluded from visualization to improve readability without affecting downstream statistical analyses.

### 2.5. Microbial Diversity Assessment and Statistical Analysis

Alpha diversity was evaluated using Observed Features, Shannon diversity, and Pielou’s evenness indices. Comparisons among sampling time points were performed using the non-parametric Kruskal–Wallis test.

Although the study followed a repeated-measures design, the small sample size limited the use of more complex longitudinal models. Therefore, results were interpreted cautiously and primarily as exploratory.

Beta diversity was assessed using weighted and unweighted UniFrac distance matrices and visualized through principal coordinates analysis (PCoA). Differences in microbial community structure were evaluated using PERMANOVA with 999 permutations, including individual identity as a blocking factor.

### 2.6. Functional Prediction of the Fecal Microbiome

Functional profiles of the fecal microbiota were inferred using PICRUSt2 v2.5.4 [25] based on 16S rRNA gene data. These predictions represent putative metabolic potential rather than direct measurements of gene expression or functional activity and were therefore interpreted with caution.

All statistical analyses were conducted in R v4.5.1 [26] using the Microeco [27] and MicrobiotaProcess [28] packages. Data visualization was performed using phyloseq [29] and ggplot2 [30].

## 3. Results

### 3.1. Bromatological Composition of the Forages

The bromatological characterization of *Medicago sativa* (captive diet) and *Brachiaria mutica* (natural forage) revealed clear differences in nutritional composition. *M. sativa* exhibited a higher crude protein content (23.25%) compared to *B. mutica*, which showed lower protein levels (9.43–13.95%) and relatively higher fiber fractions depending on plant maturity stage. Both forage types presented low lipid content and substantial fiber levels (Table 1).

For *B. mutica*, samples were analyzed at different plant developmental stages (7, 14, and 70 days) to capture variation associated with forage maturity. These values correspond to plant growth stages and should not be interpreted as equivalent to experimental sampling days.

Overall, these results provide nutritional context for interpreting fecal microbiota patterns during the transition from natural grazing conditions to a standardized alfalfa-based diet.

### 3.2. Metagenomic Profile of the Fecal Microbiota

Sequencing of the V4 region of the bacterial 16S rRNA gene generated 1,738,026 raw reads from 20 fecal samples. After quality filtering, 1,060,349 high-quality reads were retained for downstream analysis, from which 4131 amplicon sequence variants (ASVs) were identified.

Rarefaction curve analysis indicated that sequencing depth was sufficient to capture most of the microbial diversity within samples (Supplementary Figure S1). However, samples were rarefied to 500 reads per sample, corresponding to the lowest retained depth across samples. Therefore, diversity estimates should be interpreted cautiously and primarily as exploratory indicators.

### 3.3. Alpha Diversity Analysis

**Figure 2:**
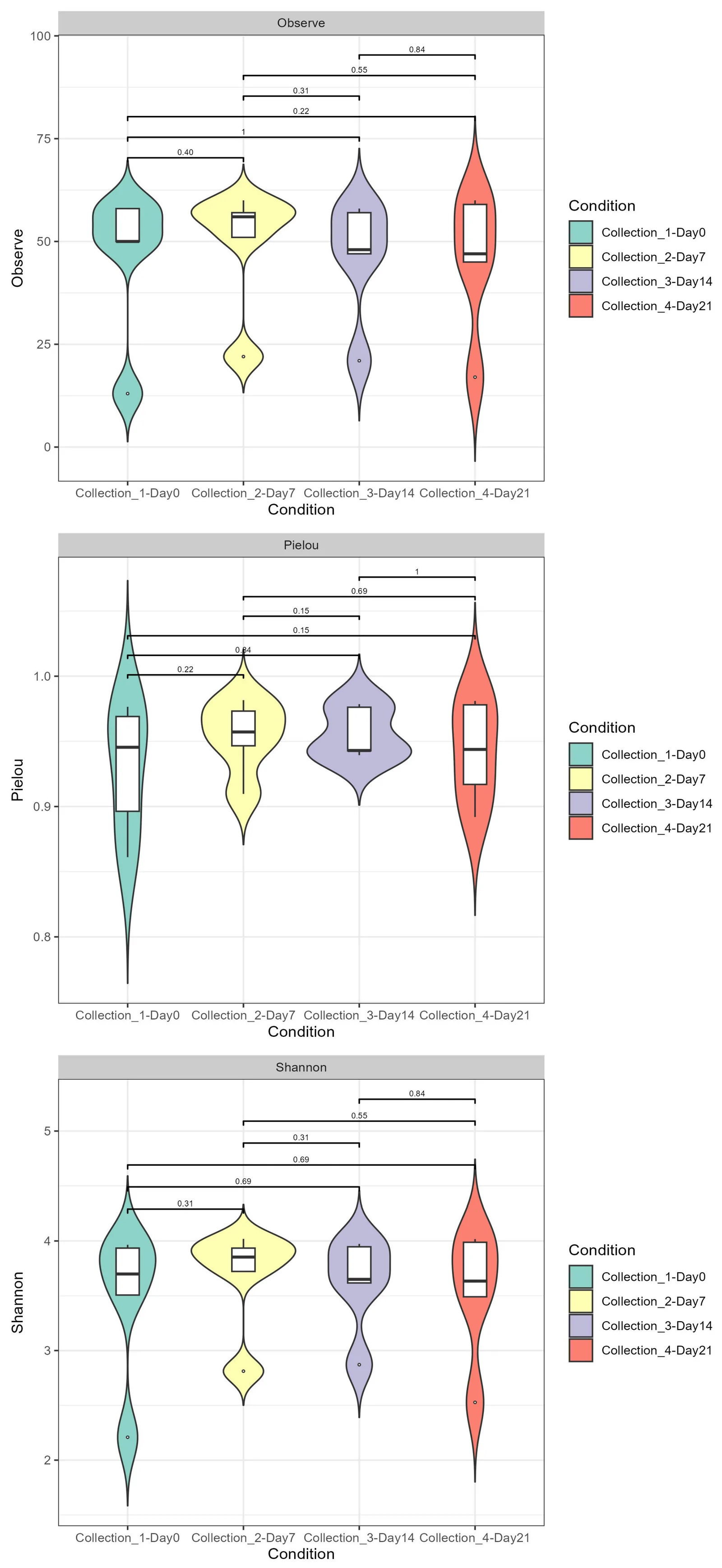
Alpha diversity analysis of the fecal microbial communities associated with *Cavia tschudii* under different collection times based on Observed Features, Pielou’s Evenness, and Shannon indices.

Alpha diversity was assessed using Observed Features, Shannon diversity, and Pielou’s evenness indices. No statistically significant differences were detected among sampling days (Day 0, Day 7, Day 14, and Day 21) for any of the evaluated metrics (Kruskal–Wallis test, *p* > 0.05; Figure 2; Supplementary Tables S2–S7).

These results indicate that fecal bacterial richness, diversity, and evenness showed limited temporal variation throughout the first three weeks of captivity.

### 3.4. Beta Diversity Analysis

**Figure 3:**
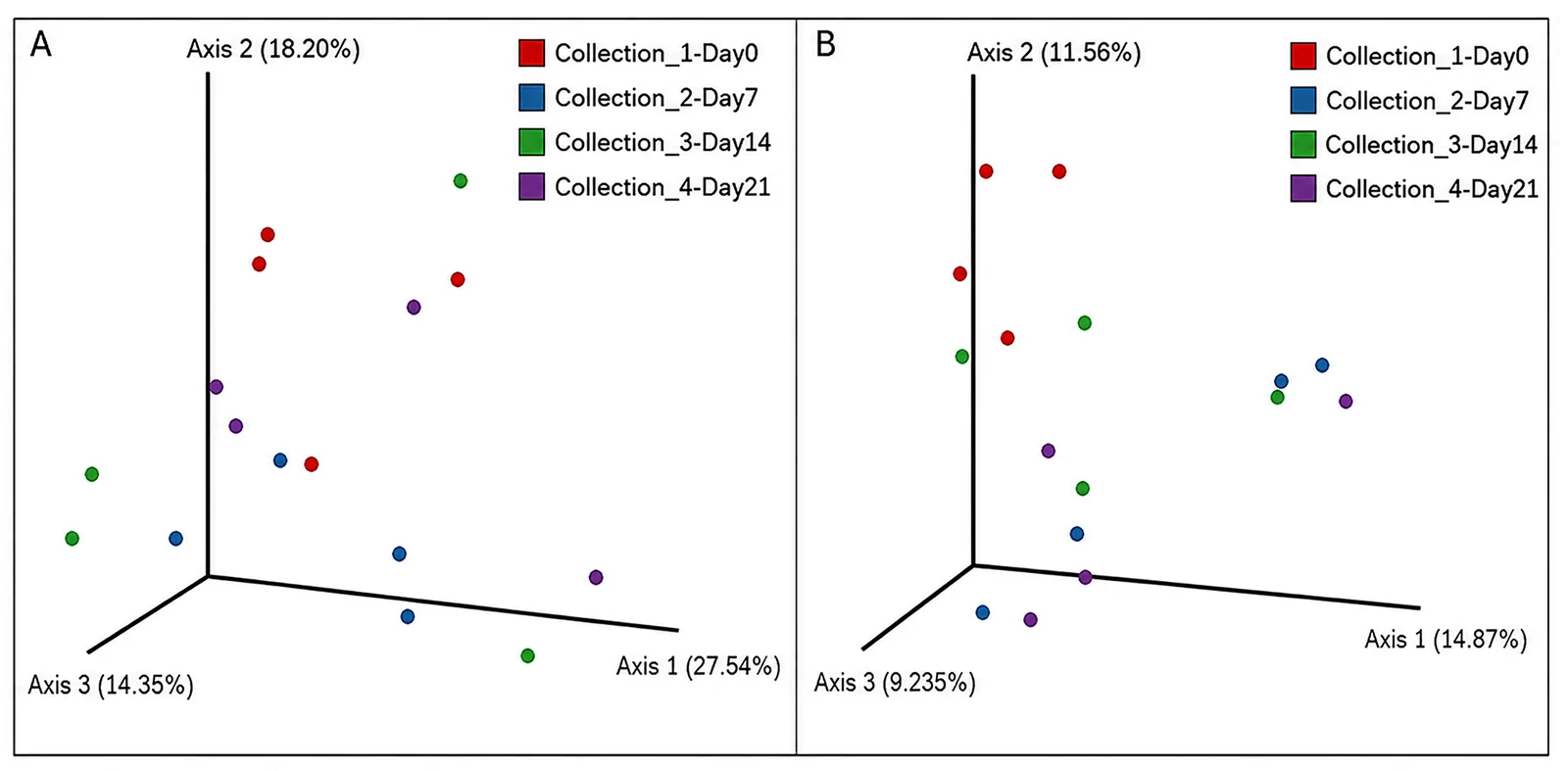
Beta diversity of the fecal microbial communities associated with *Cavia tschudii* under different collection times. Beta diversity was assessed using (**A**) weighted UniFrac distance metrics and (**B**) unweighted UniFrac distance metrics and visualized through principal coordinate analysis (PCoA). The PCoA plots illustrate the differences in microbial community composition among samples collected at Day 0 (Collection_1), Day 7 (Collection_2), Day 14 (Collection_3), and Day 21 (Collection_4). Each point represents an individual sample, and the spatial separation between points reflects differences in microbial community structure across sampling times.

Beta diversity was evaluated using weighted and unweighted UniFrac distance matrices and visualized through principal coordinates analysis (PCoA) (Figure 3). The ordination plots did not show clear clustering of samples according to sampling day. Consistently, PERMANOVA analysis did not detect statistically significant differences in microbial community structure among time points after false discovery rate correction (q > 0.05; Supplementary Table S8), even when accounting for repeated measures through individual-level blocking.

Although no statistically significant differences were detected after multiple testing correction, some comparisons exhibited marginal q-values, suggesting potential subtle temporal trends that could not be fully resolved due to the limited sample size and low sequencing depth. Therefore, the observed patterns should be interpreted cautiously, and further studies with larger sample sizes are needed to confirm these observations.

### 3.5. Taxonomic Composition

**Figure 4:**
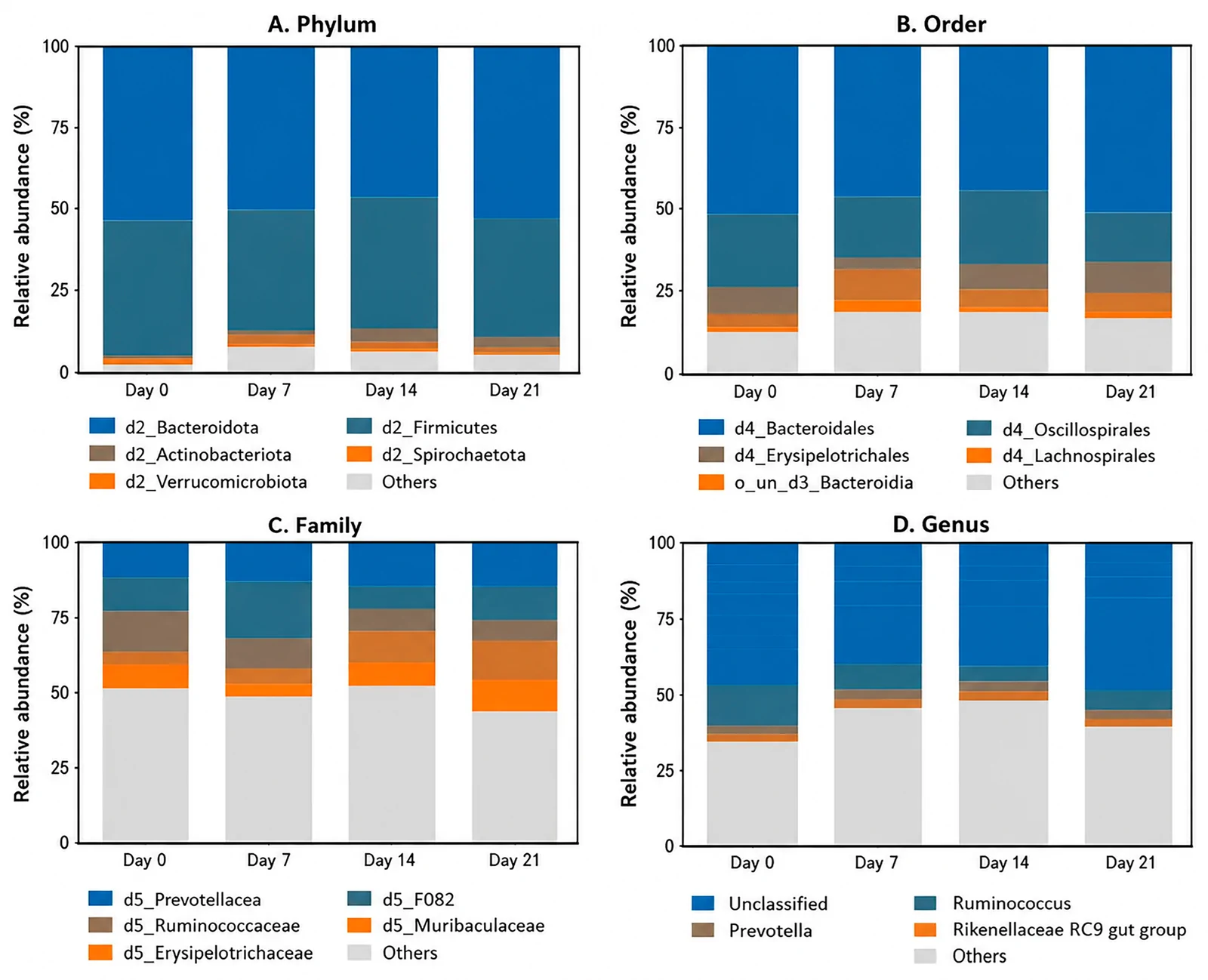
Relative abundance of the dominant bacterial taxa at the phylum (**A**), order (**B**), family (**C**), and genus (**D**) levels across experimental groups. Bar plots were generated using the ggbartax function implemented in the MicrobiotaProcess package with topn = 5, displaying the five most abundant taxa at each taxonomic level. All remaining taxa were grouped into the category Others. Family- and genus-level visualizations were generated from the curated dataset, in which unclassified placeholder taxa lacking informative taxonomic annotation were removed to improve figure readability.

The fecal bacterial community was characterized at multiple taxonomic levels (Figure 4). At the phylum level, the microbiota was consistently dominated by Bacteroidota and Firmicutes, which together accounted for more than 85% of the total relative abundance across all sampling days.

At the order level, Bacteroidales and Oscillospirales were the most abundant taxa throughout the study period. Minor bacterial orders, including Methanobacteriales and Spirochaetales, were detected at low relative abundances.

At the family level, Prevotellaceae was the predominant family across samples, followed by Ruminococcaceae and Muribaculaceae. At the genus level, members of Prevotellaceae and Muribaculaceae remained consistently among the most abundant taxa across all time points.

### 3.6. Functional Prediction of the Fecal Microbiota

Predicted functional profiles of the fecal microbiota revealed that the most abundant metabolic pathways were primarily associated with amino acid biosynthesis, nucleotide biosynthesis, and central carbon metabolism. Among the ten MetaCyc pathways with the highest relative abundance were L-isoleucine biosynthesis (PWY-5101 and PWY-5104), UMP biosynthesis (PWY-5686), 5-aminoimidazole ribonucleotide biosynthesis (PWY-6122), de novo adenosine ribonucleotide biosynthesis (PWY-7219), pyrimidine nucleobase salvage (PWY-7208), as well as pathways related to glycolysis III, cis-vaccenate biosynthesis, anaerobic gondoate biosynthesis, and the pentose phosphate pathway.

**Figure 5:**
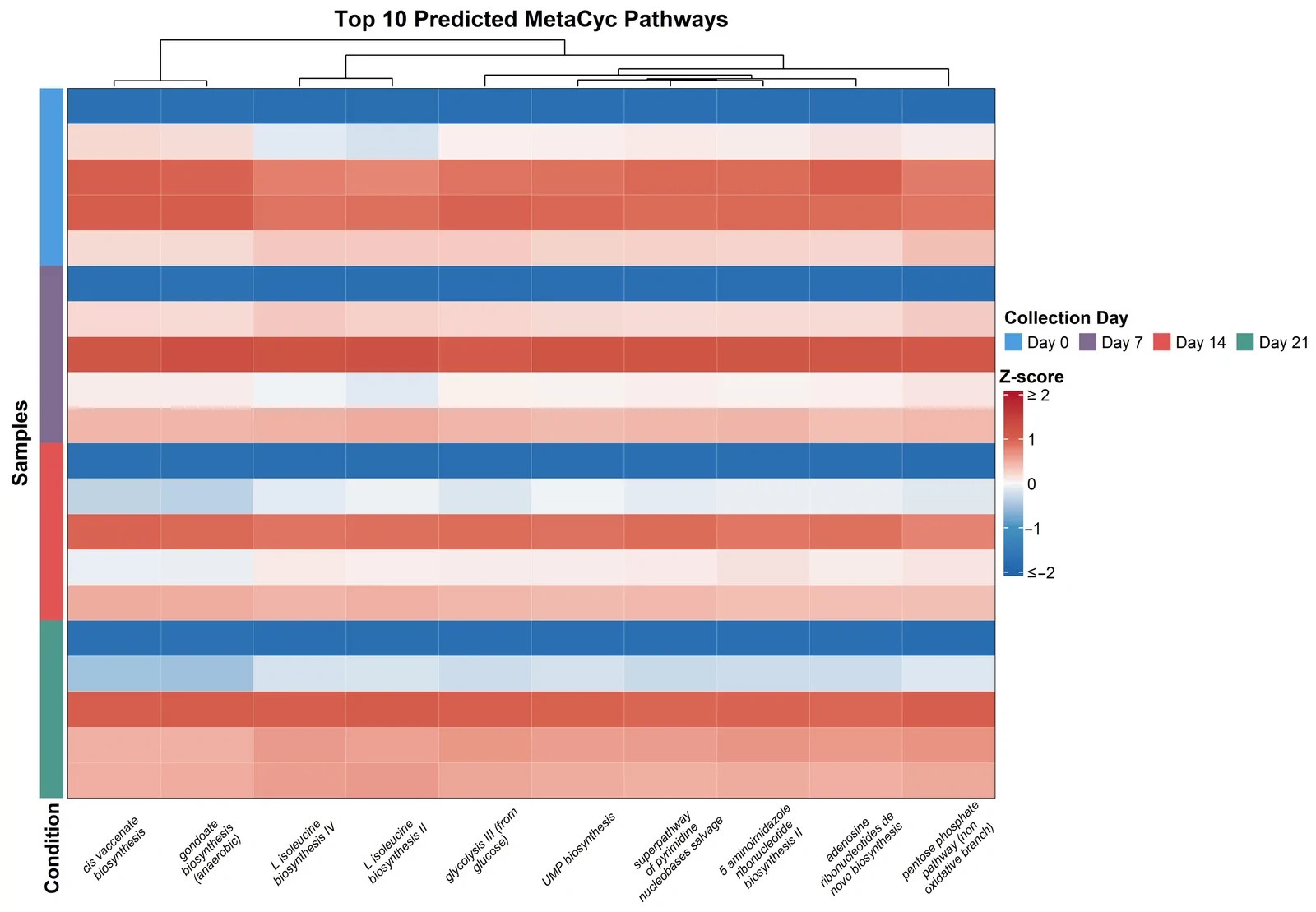
Heatmap of the top 10 MetaCyc metabolic pathways predicted by PICRUSt2 in the fecal microbiota of *Cavia tschudii* sampled longitudinally on Days 0, 7, 14, and 21. Columns represent individual samples, whereas rows correspond to the predicted metabolic pathways. Colors indicate standardized relative abundance (Z-score), and the upper dendrogram shows the hierarchical clustering based on the similarity of functional profiles.

The heatmap analysis revealed a high similarity in predicted functional profiles among samples collected on Days 0, 7, 14, and 21, with no clear clustering according to sampling time. The similarity in predicted functional profiles was further supported by statistical analyses, which did not detect significant differences in the relative abundance of any of the evaluated metabolic pathways (ANOVA, *p* = 0.997; Kruskal–Wallis, *p* = 0.985). Likewise, pairwise comparisons among sampling times showed no significant differences (*p* > 0.05), with very small effect sizes (η^2^ = 0.003–0.012) and minimal changes in pathway abundance between Day 21 and Day 0 (log2FC ranging from −0.16 to +0.05; Fold Change = 0.89–1.04). Collectively, these results suggest that the predicted functional potential of the fecal microbiota showed limited temporal variation throughout the study period (Figure 5, Supplementary Table S9).

## 4. Discussion

The present study evaluated the short-term response of the fecal microbiota of *Cavia tschudii* during its transition from natural wild conditions to captivity under a standardized alfalfa-based diet. Despite the abrupt environmental and dietary shift, no statistically detectable changes were observed in alpha diversity, beta diversity, or dominant taxonomic composition during the first 21 days. These findings suggest limited temporal variation in the fecal microbiota of *Cavia tschudii* during early captive adaptation under the conditions evaluated.

Rarefaction analysis of the 16S rRNA V4 region reached a saturation plateau, supporting that sequencing depth was sufficient to capture most of the microbial diversity present in the samples. However, because data were normalized to the minimum retained sequencing depth, diversity estimates should be interpreted cautiously. Similar approaches have been used in microbiota studies of rodents and guinea pigs to assess microbial responses to environmental and dietary variation [31,32].

The digestive physiology of caviids provides a useful framework for interpreting these results. As hindgut fermenters, guinea pigs rely on complex microbial communities specialized in the degradation of plant-derived substrates [33]. This fermentative system may contribute to maintaining microbial communities with limited temporal variation when animals are exposed to short-term environmental or dietary changes.

Bromatological analysis confirmed clear nutritional differences between *Medicago sativa* and *Brachiaria mutica*, particularly in protein and fiber content. Diet composition is widely recognized as a major driver of gut microbial structure in herbivorous mammals, as it determines the availability of fermentable substrates [34,35]. Despite the marked nutritional differences between the natural and captive diets, the transition from wild grazing to captivity did not result in significant changes in microbial diversity or overall community structure.

Coprological analysis confirmed that all individuals were free of detectable parasitic infections. This reduces the likelihood that parasite-associated alterations influenced microbial composition and strengthens the interpretation that the observed patterns reflect host–microbiota dynamics under the given environmental conditions [31,36].

Alpha diversity remained consistent across all sampling time points, indicating limited temporal variation in bacterial richness, evenness, and overall diversity. Similar patterns have been reported in rodents and other herbivorous mammals, where short-term environmental or dietary changes do not necessarily result in detectable shifts in within-sample diversity [33,34,37]. Although the limited sample size may reduce statistical power, the consistency of these indices across time points suggests limited temporal variation during the study period.

Beta diversity analyses based on UniFrac distances did not reveal significant differences in microbial community structure among sampling days after multiple-comparison correction. Although ordination plots suggested some degree of dispersion, these patterns were not statistically supported. Similar transitional responses—characterized by modest variation without strong statistical significance—have been reported in rodents exposed to environmental and dietary changes [31,37,38].

At the taxonomic level, the fecal microbiota was consistently dominated by Bacteroidota and Firmicutes, which together accounted for more than 85% of total relative abundance. These phyla are commonly dominant in herbivorous mammals and are associated with the degradation of complex carbohydrates and plant-derived polysaccharides [35,39]. The persistence of these dominant groups indicates that the functional core of the microbial community remained conserved during the transition to captivity.

Minor taxa such as Methanobacteriales and Spirochaetales were detected at low relative abundances. Although these groups have been associated with fiber fermentation and hydrogen metabolism [40,41], their low abundance and lack of significant variation suggest that they did not play a major role in shaping overall community dynamics during the study period [42,43].

Functional predictions using PICRUSt2 indicated that the predicted functional potential of the fecal microbiota showed limited temporal variation throughout the study period, with no significant differences in the relative abundance of the evaluated MetaCyc pathways. However, these predictions should be interpreted cautiously because they are based on 16S rRNA gene profiles and do not represent direct measurements of microbial activity [44,45].

Several limitations should be considered. The absence of a parallel wild control group prevents direct attribution of the observed patterns exclusively to captivity. The small sample size (*n* = 5) and low sequencing depth limit the statistical power of the study and may have reduced the ability to detect subtle temporal changes in microbial composition. Therefore, these findings should be interpreted as preliminary rather than definitive. In addition, rarefaction to a low sequencing depth may have limited the detection of low-abundance taxa and subtle shifts in community composition. Furthermore, the use of 16S rRNA sequencing restricts functional interpretation, as it provides taxonomic resolution but not direct evidence of metabolic activity.

All individuals received topical antiparasitic treatment prior to captivity. Although pyrethroid compounds have been reported to influence gut microbial communities under certain exposure routes [46], the application method and controlled conditions in this study likely minimized systemic exposure. Nevertheless, this factor should be considered when interpreting initial microbial composition.

Additionally, individuals of both sexes were included without balanced representation, and sex-related effects were not evaluated. Future studies with larger and balanced populations will be necessary to assess potential sex-related variation in fecal microbiota.

These findings provide initial evidence of limited temporal variation in the fecal microbiota of a wild caviid species under early captive conditions and contribute to a better understanding of host–microbiome interactions during environmental transitions relevant to conservation and potential domestication processes.

## 5. Conclusions

The fecal microbiota of *Cavia tschudii* did not show statistically significant changes in alpha diversity, beta diversity, or dominant taxonomic composition during the first three weeks of captivity under a standardized alfalfa-based diet. These findings suggest limited temporal variation in the gut microbial community during the study period. However, given the exploratory nature of the study, the small sample size, the low sequencing depth, and the absence of a parallel wild control group, the results should be interpreted with caution.

This study provides baseline information on microbiota dynamics in a wild caviid species and contributes to understanding host–microbiome interactions during early captive adaptation. These insights may support future research on captive management, conservation strategies, and the potential domestication of *Cavia tschudii* under controlled conditions.

## Figures and Tables

**Table 1:** Bromatological composition of forages associated with the natural and captive diets of *Cavia tschudii*.

Sample	Moisture (%)	Ash (%)	Crude Fat (%)	Protein (%)	Fiber (%)	NFE (%)
*Medicago sativa* (0 days)	5.21	9.35	2.55	23.25	27.76	31.89
*Brachiaria mutica* (7 days)	8.11	11.47	1.83	13.95	12.59	52.05
*Brachiaria mutica* (14 days)	7.3	12.89	1.51	12.82	28.22	37.25
*Brachiaria mutica* (70 days)	7.56	11.61	1.39	9.43	32.63	37.39

Note: NFE = Nitrogen-Free Extract. Days correspond to plant growth stages of *B. mutica* and not to the duration of the animal experiment.

## Data Availability

The raw sequences generated in this study were deposited in the NCBI BioProject repository under accession number PRJNA1333648.

## Supplementary Materials

The following supporting information can be downloaded at: https://www.mdpi.com/article/10.3390/microorganisms14081629/s1, Figure S1: Rarefaction curves generated using the ACE, Chao1, and Observed Features indices for the 20 processed fecal samples, obtained with the MicrobiotaProcess package. Table S1: Analytical methods and formulas used for the proximate (bromatological) analysis of forages. Table S2: Statistical significance assessed by the Kruskal–Wallis test for alpha diversity analysis using the Shannon index. Table S3: Pairwise Kruskal–Wallis test results for alpha diversity analysis using the Shannon index. Table S4: Statistical significance assessed by the Kruskal–Wallis test for alpha diversity analysis using the Pielou evenness index. Table S5: Pairwise Kruskal–Wallis test results for alpha diversity analysis using the Pielou evenness index. Table S6: Statistical significance assessed by the Kruskal–Wallis test for alpha diversity analysis using the Observed Features index. Table S7: Pairwise Kruskal–Wallis test results for alpha diversity analysis using the Observed Features index. Table S8: PERMANOVA results based on Bray–Curtis, Jaccard, Unweighted UniFrac, and Weighted UniFrac distance matrices. Table S9: Relative abundance and statistical analysis of the top 10 MetaCyc metabolic pathways predicted by PICRUSt2 in the fecal microbiota of five wild guinea pigs (*Cavia tschudii*) evaluated longitudinally on Days 0, 7, 14, and 21, including mean ± standard deviation, ANOVA, Kruskal–Wallis test, multiple comparisons, effect size (η^2^), log_2_ fold change, and fold change (Day 21/Day 0).

## References

[1] Pereira A.M., Clemente A. (2021). Dogs’ Microbiome From Tip to Toe. Top. Companion Anim. Med..

[2] Suchodolski J.S. (2022). Analysis of the gut microbiome in dogs and cats. Vet. Clin. Pathol..

[3] Gavzy S.J., Kensiski A., Lee Z.L., Mongodin E.F., Ma B., Bromberg J.S. (2023). Bifidobacterium mechanisms of immune modulation and tolerance. Gut Microbes.

[4] Xiao Y., Zhai Q., Zhang H., Chen W., Hill C. (2021). Gut colonization mechanisms of *Lactobacillus* and *Bifidobacterium*: An argument for personalized designs. Annu. Rev. Food Sci. Technol..

[5] Wang W., Dong H., Chang X., Chen Q., Wang L., Chen S., Chen L., Wang R., Ge S., Wang P. (2024). Bifidobacterium lactis and Lactobacillus plantarum enhance immune function and antioxidant capacity in cats through modulation of the gut microbiota. Antioxidants.

[6] Wei J., Wei L., Ullah A., Geng M., Zhang X., Wang C., Khan M.Z., Wang C., Zhang Z. (2025). Metagenomic applications to herbivore gut microbiomes: A comprehensive review of microbial diversity and host interactions. Animals.

[7] Belanche A., Belzecki G., Hernandez-Sanabria E., Martínez-Fernandez G., Ramos-Morales E., de la Fuente G. (2025). Editorial: Unravelling the unknown of the rumen microbiome: Implications for animal health, productivity, and beyond. Front. Microbiol..

[8] Ley R.E., Hamady M., Lozupone C., Turnbaugh P.J., Ramey R.R., Bircher J.S., Schlegel M.L., Tucker T.A., Schrenzel M.D., Knight R. (2008). Evolution of mammals and their gut microbes. Science.

[9] Chen S., Luo S., Yan C. (2022). Gut microbiota implications for health and welfare in farm animals: A review. Animals.

[10] Pacheco-Torres I., García-De la Peña C., Meza-Herrera C.A., Vaca-Paniagua F., Díaz-Velásquez C.E., Méndez-Catalá C.F., Flores-Mariñelarena A., Hernández-Martínez J., Vázquez-Díaz J., Mellado M. (2022). Exploring bovine fecal bacterial microbiota in the Mapimi Biosphere Reserve, Northern Mexico. Rev. Mex. Cienc. Pecu..

[11] Reese A.T., Chadaideh K.S., Diggins C.E., Schell L.D., Beckel M., Callahan P., Ryan R., Zambrano-Vera K., Crandall K.A., Dunn R.R. (2021). Effects of domestication on the gut microbiota parallel those of human industrialization. eLife.

[12] Integrated Taxonomic Information System (ITIS) (1867). *Cavia tschudii* Fitzinger. https://www.itis.gov.

[13] Goicochea-Vargas J., Salvatierra-Alor M., Acosta-Pachorro F., Rondón-Jorge W., Herrera-Briceño A., Morales-Parra E., Mialhe E. (2024). Genomic characterization and probiotic potential of lactic acid bacteria isolated from feces of guinea pig (*Cavia porcellus*). Open Vet. J..

[14] AOAC International (2016). Official Methods of Analysis of AOAC International.

[15] AOAC International (2019). Official Methods of Analysis of AOAC International.

[16] ANKOM Technology (2021). Analytical Methods for Fiber Analysis.

[17] Straub D., Blackwell N., Langarica-Fuentes A., Peltzer A., Nahnsen S., Kleindienst S. (2020). Interpretations of Environmental Microbial Community Studies Are Biased by the Selected 16S rRNA (Gene) Amplicon Sequencing Pipeline. Front. Microbiol..

[18] Di Tommaso P., Chatzou M., Floden E.W., Barja P.P., Palumbo E., Notredame C. (2017). Nextflow enables reproducible computational workflows. Nat. Biotechnol..

[19] Andrews S. (2010). FastQC: A Quality Control Tool for High Throughput Sequence Data.

[20] Ewels P., Magnusson M., Lundin S., Käller M. (2016). MultiQC: Summarize analysis results for multiple tools and samples in a single report. Bioinformatics.

[21] Martin M. (2011). Cutadapt removes adapter sequences from high-throughput sequencing reads. EMBnet. J..

[22] Callahan B.J., McMurdie P.J., Rosen M.J., Han A.W., Johnson A.J.A., Holmes S.P. (2016). DADA2: High-resolution sample inference from Illumina amplicon data. Nat. Methods.

[23] Bolyen E., Rideout J.R., Dillon M.R., Bokulich N.A., Abnet C.C., Al-Ghalith G.A., Alexander H., Alm E.J., Arumugam M., Asnicar F. (2019). Reproducible, interactive, scalable and extensible microbiome data science using QIIME 2. Nat. Biotechnol..

[24] Quast C., Pruesse E., Yilmaz P., Gerken J., Schweer T., Yarza P., Peplies J., Glöckner F.O. (2013). The SILVA ribosomal RNA gene database project: Improved data processing and web-based tools. Nucleic Acids Res..

[25] Douglas G.M., Maffei V.J., Zaneveld J.R., Yurgel S.N., Brown J.R., Taylor C.M., Huttenhower C., Langille M.G.I. (2020). PICRUSt2: An improved and extensible approach for metagenome inference. Nat. Biotechnol..

[26] R Core Team (2025). R: A Language and Environment for Statistical Computing, Version 4.5.1.

[27] Liu C., Cui Y., Li X., Yao M. (2021). Microeco: An R package for data mining in microbial community ecology. FEMS Microbiol. Ecol..

[28] Xu S., Yu G., Hu T. (2023). MicrobiotaProcess: An R package for analysis, visualization and biomarker discovery of microbiome. Bioinformatics.

[29] McMurdie P.J., Holmes S. (2013). Phyloseq: An R Package for Reproducible Interactive Analysis and Graphics of Microbiome Census Data. PLoS ONE.

[30] Wickham H. (2016). ggplot2: Elegant Graphics for Data Analysis.

[31] Anders S., Figueroa C., Vega M. (2021). Gut microbiota dynamics in rodents under environmental and dietary shifts. Front. Microbiol..

[32] Viney M. (2019). Ecological and evolutionary dynamics of host-microbiota interactions in wild rodents. Trends Ecol. Evol..

[33] Noonan D.E. (1994). The Guinea Pig (*Cavia porcellus*). ANZCCART.

[34] Figueroa C., Frias D., Anders S. (2021). Effects of diet and environment on intestinal microbiota of wild rodents. BMC Genom..

[35] Kamareddine L., Najjar H., Sohail M.U., Abdulkader H., Al-Asmakh M. (2020). The Microbiota and Gut-Related Disorders: Insights from Animal Models. Cells.

[36] Cantaro Segura J., Cántaro-Segura H., Blas R. (2026). 16S rRNA Metagenomic Profiling Reveals Diet-Induced Shifts in Gut Microbial Diversity and Taxonomic Structure in Guinea Pigs. Appl. Microbiol..

[37] Cuenca-Condoy M., Campos-Murillo N., Quinteros-Rodas W., Miranda-Yuquilema J. (2026). Guinea pigs in balance: Impact of the probiotic diet on the pH, microbiota and productive performance of guinea pigs. Front. Anim. Sci..

[38] Wu Y., Fan H., Feng Y., Yang J., Cen X., Li W. (2024). Unveiling the gut microbiota and metabolite profiles in guinea pigs with form deprivation myopia through 16S rRNA gene sequencing and untargeted metabolomics. Heliyon.

[39] Frias H., Murga Valderrama N.L., Flores Durand G.J., Cornejo V.G., Romani A.C., Bardales W., Segura G.T., Polveiro R.C., Vieira D.S., Ramos Sanchez E.M. (2023). Comparative analysis of fasting effects on the cecum microbiome in three guinea pig breeds: Andina, Inti, and Peru. Front. Microbiol..

[40] Volmer J.G., McRae H., Morrison M. (2023). The evolving role of methanogenic archaea in mammalian microbiomes. Front. Microbiol..

[41] Cisek A.A., Szymańska E., Aleksandrzak-Piekarczyk T., Cukrowska B. (2024). The Role of Methanogenic Archaea in Inflammatory Bowel Disease—A Review. J. Pers. Med..

[42] Thingholm L.B., Bang C., Rühlemann M.C., Starke A., Sicks F., Kaspari V., Jandowsky A., Frölich K., Ismer G., Bernhard A. (2021). Ecology impacts the decrease of Spirochaetes and Prevotella in the fecal gut microbiota of urban humans. BMC Microbiol..

[43] Tokuda G., Mikaelyan A., Fukui C., Matsuura Y., Watanabe H., Fujishima M., Brune A. (2018). Fiber-associated spirochetes are major agents of hemicellulose degradation in the hindgut of wood-feeding higher termites. Proc. Natl. Acad. Sci. USA.

[44] Putri S.S.F., Irfannuddin I., Murti K., Kesuma Y., Darmawan H., Koibuchi N. (2023). The role of gut microbiota on cognitive development in rodents: A meta-analysis. J. Physiol. Sci..

[45] de Miguel M., de Maria N., Guevara M.Á., Diaz L., Sáez-Laguna E., Sánchez-Gómez D., Chancerel E., Aranda I., Collada C., Plomion C. (2012). Annotated genetic linkage maps of *Pinus pinaster* Ait. from a Central Spain population using microsatellite and gene based markers. BMC Genom..

[46] Yue S., Yuan Q., Shen Q., Xu Y., Wang P., Si M., Zhao M. (2023). Multiomics implicate gut microbiota in low cypermethrin exposure induced multiorgan toxicological effects in pubertal male rats. J. Hazard. Mater..

