# Supplementary material for: Short-Term Stability of the Fecal Microbiota in Wild Cavia tschudii During Early Captivity Transition
Source: Microorganisms. 2026 Jul 26;14(8):1629. doi: 10.3390/microorganisms14081629 (PMC13515332; doi:10.3390/microorganisms14081629)
Supplement: Supplementary file 1 [file microorganisms-14-01629-s001.zip › MATERIAL SUPLEMENTARIO - FIGURA S1.pdf]

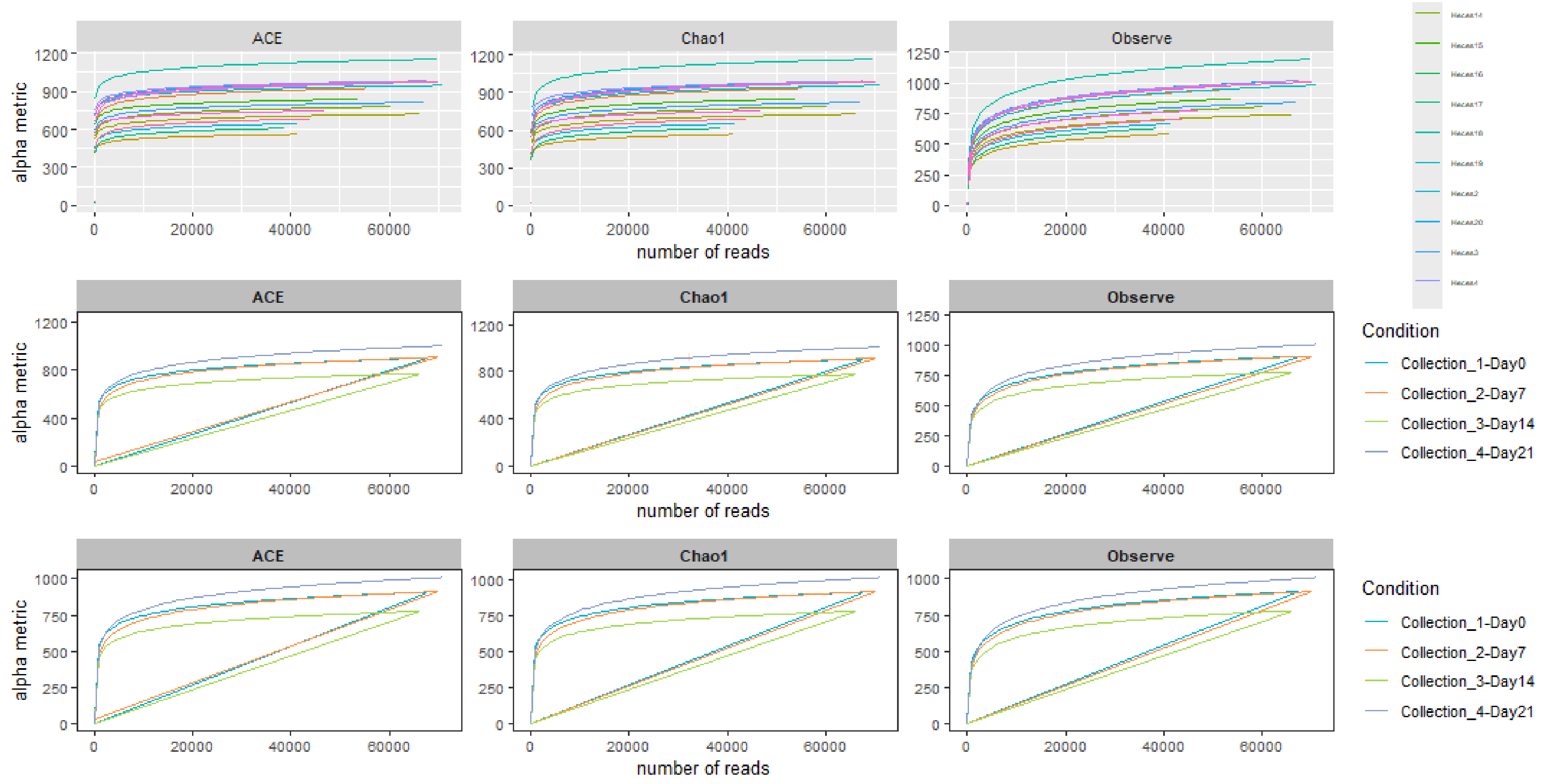

Figure S1: Rarefaction curves generated using the ACE, Chao1, and Observed Features indices for the 20 processed fecal samples, obtained with the MicrobiotaProcess package.
